# Large-scale otoscopic and audiometric population assessment: A pilot study^☆^

**DOI:** 10.1016/j.ijporl.2018.11.033

**Published:** 2019-02

**Authors:** Kenny H. Chan, Susan Dreith, Kristin M. Uhler, Veronica Tallo, Marilla Lucero, Joanne De Jesus, Eric AF. Simões

**Affiliations:** a Department of Otolaryngology-Head and Neck Surgery, University of Colorado School of Medicine, Aurora, CO, United States; b Department of Pediatric Otolaryngology, Children's Hospital Colorado, Aurora, CO, United States; c Department of Pediatrics, University of Colorado School of Medicine, Aurora, CO, United States; d Private Practice Audiologist, Denver, CO, United States; e Department of Physical Medicine and Rehabilitation, University of Colorado School of Medicine, Department of Audiology, Speech-Pathology, and Learning Children's Hospital Colorado, Aurora, CO, United States; f Department of Clinical Trials, Epidemiology and Biostatistics, Research Institute for Tropical Medicine Department of Health, Muntinlupa, Manila, Philippines; g Department of Epidemiology Center for Global Health, Colorado School of Public Health, Aurora, CO, United States

**Keywords:** Otoscopic assessment, Audiometric assessment, Otitis media

## Abstract

**Objective:**

Large-scale otoscopic and audiometric assessment of populations is difficult due to logistic impracticalities, particularly in low- and middle-income countries (LMIC). We report a novel assessment methodology based on training local field workers, advances in audiometric testing equipment and cloud-based technology.

**Methods:**

Prospective observational study in Bohol, Philippines. A U.S. otolaryngologist/audiologist team trained 5 local nurses on all procedures in a didactic and hands-on process. An operating otoscope (Welch-Allyn^R^) was used to clear cerumen and view the tympanic membrane, images of which were recorded using a video otoscope (JedMed^R^). Subjects underwent tympanometry and distortion product otoacoustic emission (DPOAE) (Path Sentiero^R^), and underwent screening audiometry using noise cancelling headphones and a handheld Android device (HearScreen^R^). Sound-booth audiometry was reserved for failed subjects. Data were uploaded to a REDCap database. Teenage children previously enrolled in a 2000–2004 Phase 3 pneumococcal conjugate vaccine trial, were the subjects of the trainees.

**Results:**

During 4 days of training, 47 Filipino children (M/F = 28/19; mean/median age = 14.6/14.6 years) were the subjects of the trainee nurses. After the training, all nurses could perform all procedures independently. Otoscopic findings by ears included: normal (N = 77), otitis media with effusion (N = 2), myringosclerosis (N = 5), healed perforation (N = 6), perforation (N = 2) and retraction pocket/cholesteatoma (N = 2). Abnormal audiometric findings included: tympanogram (N = 4), DPOAE (N = 4) and screening audiometry (N = 0).

**Conclusion:**

Training of local nurses has been shown to be robust and this methodology overcomes challenges of distant large-scale population otologic/audiometric assessment.

## 1 Introduction

Otitis media (OM) and its most consequential complication, chronic suppurative otitis media (CSOM), are disproportionally overrepresented in developing countries, particularly in Asia and sub-Saharan Africa [1]. The burden of disease from CSOM includes hearing loss, tympanic membrane perforation, otorrhea, cholesteatoma and intratemporal and intracranial complications which in turn have important downstream social, educational and vocational impact. OM global health initiatives and clinical research in these populations mandate accurate epidemiologic assessments in LMIC.

However, large-scale otoscopic and audiometric assessment of ear disease in children in LMIC is difficult due to logistic constraints, including a lack of portable equipment, and trained audiologists and otorlaryngologists with the time and interest for these studies. A survey [[2], [3], [4], [5], [6], [7], [8]] of published reports in the past 15 years with relevance to OM through population studies in LMIC and developing countries has revealed one or more methodologic deficiencies; namely, small sample size, lacking either otoscopic or audiometric evaluation and particularly, assessment of CSOM.

Two recent studies have overcome these apparent deficiencies including the documentation of frequencies of CSOM through large-scale population surveys in at-risk regions including Asia (Indonesia) [9] and Africa (Kenya) [10]. Yet both of these studies relied heavily on onsite and/or local highly-trained otolaryngologists and audiologic personnel. In the Indonesian study, locally trained certified audiologists conducted hearing testing. In the Kenyan study, local medical officers were utilized to carry out clinical assessments and audiometric assessments. While frequencies of CSOM were assessed, both required significant local trained audiologic and/or clinical personnel with limited Western-trained audiologist and pediatrician/otolaryngologist supervision and monitoring. Both the Indonesian and Kenyan studies were carried out by research teams visiting schools and conducting cross-sectional studies over 3–5 days at each school that lasted for several months. However, there have been no recent population-based studies that could extend beyond the confines in terms of sample size and resource commitment described in these studies.

Our research team recently received funding to assess the long-term audiometric and otologic status as well as cognitive development of a cohort of over 12,000 teenagers. They were previously enrolled in a randomized clinical trial [11] that evaluated the safety and efficacy of an 11-valent pneumococcal conjugate vaccine for radiographically confirmed pneumonia when these children were less than 2 years of age. These research subjects lived in Bohol, Philippines, an island with a land mass of 4821 km^2^ (1861 mi^2^). The enormity of this research undertaking defied all cited conventional methods. A new methodology had to be developed that would allow local research personnel to conduct these otologic and audiometric assessments using portable testing equipment that could be transported to remote municipalities on the island of Bohol and allow periodic Internet/cloud uploads to facilitate ongoing monitoring of data quality and eventual data analysis, from Manila and Denver.

With the advances in audiometric testing equipment suitable for field deployment and cloud-based technology, our team hypothesized that it was possible to develop a methodology based on new technologies to conduct this large-scale population based otoscopic and audiometric assessment in Philippines following hands-on training provided by a U.S. audiologist/otolaryngologist team for local field workers. Furthermore, we wished to demonstrate that this methodology is capable of allowing continued supervision and monitoring. This paper describes the otoscopic/audiometric methodologic aspects of the larger study through the analysis of a pilot sample.

## 2 Material and methods

IRB approval for the overall study was obtained from the Research Institute of Tropical Medicine, Manila Philippines and the COMIRB at the University of Colorado School of Medicine, Aurora, USA. The methodology is described in 4 sections: overall construct for data collection, storage and analysis; training of local field workers, the promulgation of the “do no harm” precept and continuous data quality monitoring and troubleshooting.

### 2.1 Overall construct

The overarching goal was to collect both objective (tympanometry, and distortion product otoacoustic emissions - DPOAE) and subjective (screening audiometry, still and video otoscopy images) data by trained local nurses as discussed below in Bohol, Philippines and be able to analyze them in the U.S. It was also to establish a mechanism to provide continued support for the local field workers and maintain data quality. This necessitated a robust training program for the field workers by a U.S. team as described below. It also required a willingness for the U.S. investigators to answer all queries generated by the field workers in a timely manner (within 48 h) based on uploaded data via email.

All subjects underwent otoscopy using an operating otoscope (Part #: 21700, Welch-Allyn^R^, Skaneateles Falls, NY, USA). Once the entire circumference of the tympanic membrane was in full view, images (still and video) were recorded using a video otoscope (Horus + HD Video Otoscope, #39-7405, JedMed, St. Louis, MO, USA). This unit uses an SD card for image storage in the JPEG format. Each subject then underwent tympanometry and DPOAE recordings (Sentiero//Tymp Screening SOD04, Path Medical, Germering, Germany). This unit allows tympanometry to be recorded at 266 Hz and DPOAE recorded in 4 frequencies sequentially without removal of the probe during testing. Although the unit stored the raw data, the output for the research nurses were pass/fail binary outcomes for both tympanometry and DPOAE. Data were stored within the unit until the time for download. Finally, each subject then underwent screening audiometry using noise cancelling headphones (Sennheiser HD 280 Pro, Wedemark, Germany) linked to a handheld audiometer operated through an Android device manufactured by HearScreen (Pretoria, South Africa). This unit allows pure tone frequencies from 0.5 to 15 kHz to be tested and it also generates a pass/fail binary outcome as defined as >35 dB HL at any frequency in either ear. The Maximum Permissible Ambient Noise Level (MPANL) for the amount of ambient noise permissible for audiometric testing for this system ranges from 27 dB at 500 Hz to 44 dB at 2000 Hz. The unit generates a green light when the ambient sound frequencies are below the thresholds or a red light when the thresholds are exceeded; local nurses were trained to only conduct screening audiometry when the green light was on. Binary outcome and raw data are directly uploaded through cloud technology to HearScreen.

All data with the exception of the screening audiometry data were downloaded at the local research facility first onto a personal desktop computer from their respective storage sources. Screening audiometry data were downloaded from the HearScreen cloud storage site. Backup files were made and were stored on an external hard drive. All downloaded data were then uploaded in batches to a REDCap database [12] set up at University of Colorado, School of Medicine, Aurora, CO, USA.

All otoscopic data with the aid of tympanometric data (by ear) were classified into one of the following categories: normal, otitis media with effusion, myringosclerosis, perforation, healed perforation and retraction pocket/cholesteatoma. For the sample described in this paper, the diagnoses were made by the first author (KHC). For the analysis of the complete dataset, U.S. otolaryngology advanced practice providers will be trained in the future to interpret the videootoscopic findings.

### 2.2 Field worker training

The training methodology included didactic and hands-on training of local nurses led by a U.S. otolaryngologist/audiologist team. The main objective of the nurses was to collect the data described and they themselves did not participate in diagnostic decisions. The only decisions made by them were related to referral to the local otolaryngologists and for full audiologic evaluation dictated by the research protocol. It included teaching the nurses to remove cerumen using an operating head otoscope and the use of all equipment described above. Maintenance and calibration of all equipment as well as the storage and transfer of all data were taught. Written standard operating procedures were developed to ensure standards are kept in case of personnel changes. They included referral criteria to Bohol Hearing Center and to the local otolaryngologist for otorrhea.

### 2.3 “Do no harm” precept

Accommodations were made in the methodology to refer the research subjects to local healthcare professionals. The local field workers were instructed to refer subjects to Bohol Hearing Center for failed DPOAEs, tympanometry, and/or screening audiometry and to the local otolaryngologist for ear pathology (inability to remove cerumen after instillation of mineral oil from a previous visit, otorrhea and suspicion for cholesteatoma).

### 2.4 Ongoing training, data monitoring and troubleshooting

The nurses rotate their duties on a weekly basis, both to build repetitiveness and to minimize boredom. The supervisory nurse is responsible for coordination of all study subjects and backing up of all of the data on a daily basis. Weekly quality control monitoring is done by a data manager onsite and periodic quality checks by one of the investigators at every site visit through the past year.

Ongoing training of the field workers is achieved by the U.S. investigators monitoring the data quality through periodic REDCap database sampling. Queries generated by the field workers are resolved by the U.S. investigators via email.

## 3 Results

A U.S. otolaryngologist/audiologist team provided a 1-day instruction for 5 Filipino nurses through didactic lectures on OM and basics in audiology as well as hands-on training for all otoscopic and audiometric procedures. On-the-job training continued for the subsequent 3 days. 47 Filipino children (M/F = 28/19; mean/median age = 14.6/14.6 years) were enrolled and the nurses were able to perform all procedures independently with decreasing need for assistance from the instructors during the course of the 4-day period. Raw data were reviewed and categorized on site at the local research facility by the team with the nurses for teaching purposes. The identical dataset once uploaded to the RedCap was again reviewed and categorized in Colorado once the US team returned home.

Of the 94 ears in the cohort, cerumen sufficient to occlude the full peripheral view of the tympanic membrane was found in 40 ears (42.6%). Cerumen in 6 ears (3 subjects) could not be removed by the nursing team and these subjects were sent home with mineral oil. They returned the following week and all 6 ears were cleared of cerumen and completed the assessments. The diagnoses of the 94 ears were categorized in Table 1. Otoscopic findings of the 94 ears included: normal (N = 77), otitis media with effusion (N = 2), myringosclerosis (N = 5), healed perforation (N = 6), perforation (N = 2) and retraction pocket/cholesteatoma (N = 2). Abnormal audiometric findings included: tympanogram (N = 4), DPOAE (N = 4) and screening audiometry (N = 0). Three of the four subjects with either perforation or retraction pocket/cholesteatoma failed either the screening tympanometry or OAE or both. One subject with a retraction pocket had normal tympanogram and DPOAE. None failed the screening audiometry.

**Table 1:** Otoscopic and audiometric characteristics of the cohort by ears.

Characteristic	Total Ears (N = 94)
**Otoscopic Diagnosis, No**.	
Normal	77
Otitis media with effusion	2
Myringosclerosis	5
Healed perforation	6
Perforation	2
Retraction pocket/cholesteatoma	2
**Tympanogram, No.**	
Pass	90
Fail	4
**DPOAE, No.**	
Pass	90
Fail	4
**Screening Audiometry, No.**	
Pass	94
Fail	0

Per protocol the 4 subjects with abnormal audiometric findings were referred for formal audiogram. Two individuals were found to have chronic otorrhea despite being seen by the local otolaryngologist and have not had an audiogram at the time of preparation of this manuscript. The subject with a unilateral perforation was found to have a corresponding conductive hearing loss. The subject with a unilateral abnormal DPOAE was found to have normal hearing threshold bilaterally.

The number of emails between the Filipino field workers and U.S. investigators were tabulated based on a unique incident or patient from November 2016 to April 2018. These email exchanges included 3 instrument breakages, 2 instrument calibration issues, 17 research protocol-related and 16 patient-related queries. All queries were resolved satisfactorily; some required more than 1 cycle of emails.

## 4 Discussion

Prior to this effort, most large-scale studies of CSOM and its sequelae in school-aged children in LMIC have either been conducted at otolaryngology clinics, or as cross-sectional studies in schools or preschools, and rarely in population-based studies from random samples [13] or door to door surveys [14]. In the otolaryngology clinic setting, standard methods were used for otoscopy and diagnostic audiometry [[15], [16], [17], [18]]. In the school-based studies, for the most part trained otolaryngologists conducted the otoscopy [19,20] and screening audiometry was done in the schools [9,21,22]. Diagnostic audiometry, when done was rarely performed in the schools [10] but most often at a referral hospital [[23], [24], [25], [26]]. Only our prior studies used tympanometry [9,10] but DPOAE for audiometric screening was not used. All of these studies utilized otolaryngologists and or trained audiologists in the field to do the studies. While trained ear health research officers [27] and nurses [28] have been used in the past, to conduct community and school based surveys of Aboriginal Australian children using tympanometers, voroscopes and video-otoscopes, followed by later specialist review in country, our study has extended these methodologies using more robust appropriate technology that can be used in LMIC and other deve, oping countries.

In this study, we were able to use trained nurses to do most of these procedures, with the help of i) robust modern technology (video-otoscopy with digital recording; DPOAE and tympanometry and android mobile telephone based screening audiometry), ii) the ability to train nurses to record abnormalities on video-otoscopy, and automate DPOAE, tympanometry and screening audiometry results to pass or fail, and iii) and the ability to collect all of this data in a digital manner and to download all of the data in real-time on to an Internet-based database.

This study affirms the robustness of several key components of the methodology. Local Filipino nurses with proper didactic and hands-on training were capable of recording still and video images on the first pass which included cleaning 88% of the ears with cerumen. Furthermore, the nurses were able to use semi-automated audiometric equipment to obtain tympanograms, DPOAE and screening audiograms in all the 47 subjects. The ability to transfer otoscopic and audiometric data both in terms of data storage at the local research facility in the Philippines and uploading them to the REDCap database in Colorado were determined to be both feasible and reliable. These features in the methodology allowed not only data analysis of the present study but ongoing monitoring of data quality of the full project.

Although it is impossible to extrapolate the effects of the pneumococcal vaccine on the prevalence CSOM and its disease burden in the large cohort of children at this time, the random sampling of 47 children suggests that both cerumen impaction and complications of OM might both be higher than what one would expect when compared to a U.S sample in everyday clinical practice.

The major limitation of the study lies in the duration of the didactic and on-the-job training for the nurses in preparation for the data collection phase of the study. It would have been more ideal to have spent more time in the training of the nurses. But resource limitations in terms of time availability of the US trained personnel and additional monetary needs to execute a longer training program precluded this option. In a global health research environment, the authors strongly assert that ongoing support in clinical decision-making, adherence to research protocol and upkeep of research equipment has made this methodology rigorous to go forward in evaluating the larger cohort.

The other limitation of this methodology, is its dependence on fairly expensive equipment, the necessity for continuous power to run a lot of the equipment, the necessity for good high-speed Internet access to upload data especially video-otoscopic data, which on average ranges between 35 and 50 MB, and the necessity for a rapid response from an otolaryngologist to interpret video-otoscopic data. We did encounter occasional problems such as power outage, malfunction of the video otoscope and immediate availability of U.S. clinical personnel for troubleshooting. However, the major advantage of this methodology, now validated by over 4500 children having been screened in the last year with near-complete data collection on every child, outweighed these disadvantages. We propose that this methodology could be used with minimal training of nursing staff, for larger scale population-based studies that does not require intense intervention from busy local otolaryngologists in LMIC. There are only 3 otolaryngologists in Bohol servicing a population over 1.3 million and our research methodology worked well in that scenario.

A novel methodology has been developed and field-tested and is deemed sufficiently robust in assessing the otologic and audiometric status of a cohort of Filipino teenagers using advanced portable audiometric equipment and cloud technology that is currently deployed in a large-scale project. This methodology likely has applicability in other LMIC, for large-scale population based studies.

## Funding

This study was supported by the Bill & Melinda Gates Foundation (grant number OPP1142570) and Colorado CTSA Grants UL1TR002535, KL2TR002534, and TL1TR002533. The contents of this report are solely the responsibility of the authors and do not necessarily represent the official views of their institutions or organizations or of the sponsors. The funders did not participate in any aspect of the study, including study-conduct, data collection, analyses of the data or the write-up of the manuscript.

## Conflicts of interest

The authors certify that they have no conflicts of interest.
